# Genetic dissection of crown rust resistance in oat and the identification of key adult plant resistance genes

**DOI:** 10.1002/tpg2.70059

**Published:** 2025-06-13

**Authors:** Nikwan Shariatipour, Mahboobeh Yazdani, Anders Carlsson, Therése Bengtsson, Shahryar F. Kianian, Marja Jalli, Mahbubjon Rahmatov, the PPP RobOat Consortium

**Affiliations:** ^1^ Department of Plant Breeding Swedish University of Agricultural Sciences Alnarp Sweden; ^2^ USDA‐ARS Cereal Disease Laboratory Saint Paul Minnesota USA; ^3^ Natural Resources Institute Finland Jokioinen Finland

## Abstract

Crown rust (*Puccinia coronata* f. sp. *Avenae* Erikss.) poses a significant threat to oat production worldwide. The most effective strategy for managing this disease involves identifying, mapping, and deploying resistance genes to develop cultivars with enhanced resistance. In this study, we conducted a meta‐analysis of quantitative trait loci (QTLs) linked to crown rust resistance across diverse oat populations and environments. From 11 studies conducted between 2003 and 2024, we selected 167 QTLs, of which 127 were successfully mapped onto an oat consensus linkage map. These QTLs were mainly located on chromosomes of the D and C sub‐genomes, showing considerable variation in genetic distances and marker associations. Based on the integration of these QTLs in a meta‐QTL (MQTL) analysis, 23 MQTLs were identified for crown rust resistance in the oat genome. Gene mining within the MQTL intervals identified 1526 candidate genes, most of which were located in the D sub‐genome. Functional analysis revealed that these genes play key roles in stress response, hormonal regulation, and polyamine metabolism, which are crucial for plant defense. Conserved regulatory elements (*cis*‐acting regulatory element [CAREs]) were also identified in the promoter regions of key resistance genes, indicating their involvement in light response, stress regulation, and hormone signaling. This study represents a significant advancement in understanding the genetic architecture of crown rust resistance in oat and provides a valuable resource for breeding programs focused on improving disease resistance.

## Core Ideas

- For genome‐wide meta‐analysis, 167 QTLs for crown rust resistance in oat were included.
- The study identified 23 meta quantitative trait loci (MQTL) regions, which provide the basis for breeding programs to improve oat resistance to crown rust.
- Gene mining within MQTL intervals revealed 1526 candidate genes associated with stress response and defense mechanisms.
- Functional analysis identified 12 key genes involved in plant defense, hormonal regulation, and stress adaptation.
- Findings provide valuable insights into developing oat cultivars with enhanced and durable crown rust resistance.

**Abbreviations**
ABA: abscisic acid
AFLP: amplified fragment length polymorphism
AIC: Akaike information criterion
APR: adult plant resistance
CAREs: *cis*‐acting regulatory element
CI: confidence interval
CRs: crown rust severity
DS: disease severity
GA: gibberellic acid
GO: gene ontology
JA: jasmonic acid
LOD: logarithm of odds
MAS: marker‐assisted selection
MeJA: methyl jasmonate
MQTL: meta quantitative trait locus
PAs: polyamines
*Pc*
: *Puccinia coronata*
*Pca*
: *Puccinia coronata* f.sp. *avenae* Erikss.
PVE: phenotypic variation explained
QTL: quantitative trait locus
RFLP: restriction fragment length polymorphism

## 1 INTRODUCTION

Crown rust, caused by the fungus *Puccinia coronata* f.sp. *avenae* Erikss. (*Pca*), is a major fungal pathogen of oat, threatening global oat production by significantly reducing yield and quality. This disease affects various oat species, such as *Avena sativa* L. (AACCDD, 2*n* = 6*x* = 42), *Avena sterilis* (AACCDD, 2*n* = 6*x* = 42), *Avena strigosa* (AsAs, 2*n* = 2*x* = 14), *Avena barbata* (AABB, 2*n* = 4*x* = 28), and so on (Chong et al., 2011). Yield losses in severe outbreaks can exceed 50%, creating a substantial economic burden for oat producers (McNish et al., 2020; Nazareno et al., 2018). The increasing cultivation of oat in Europe and the Nordic countries has corresponded with a rise in crown rust severity (CRs) (Marshall et al., 2013; Vilvert et al., 2021), largely due to the expansion of oat‐growing areas and the presence of alternate hosts, such as buckthorn, which create favorable conditions for pathogen survival and evolution (Berlin et al., 2018). Additionally, changes in environmental conditions are projected to increase the severity and frequency of crown rust outbreaks in more geographical locations, further threatening oat production (Kim et al., 2024).

The complex interaction between host availability and pathogen genetic diversity is central to developing new virulent pathogen races capable of overcoming existing resistance in oat cultivars (Nazareno et al., 2018). Sexual reproduction facilitated by alternate hosts like buckthorn increases pathogen genetic diversity, which allows for the emergence of novel combinations of pathogenicity genes (Simons, 1985). In response to the threat posed by crown rust, oat breeding programs have focused on developing resistant varieties through the identification and deployment of resistance genes, particularly race‐specific genes known as *R* genes. Over 100 such resistance genes (*Puccinia coronata* [*Pc*] genes) have been identified in species like *A. sativa*, *A. strigosa*, *Avena byzantina*, and *A. sterilis* (Klos et al., 2017; McMullen et al., 2005; Park et al., 2022). These genes follow a gene‐for‐gene interaction model, where resistance is conferred by specific interactions between host resistance genes and corresponding virulent genes in the pathogen (Flor, 1955). However, the high genetic variability of the *Pc* (Park et al., 2022) undermines the durability of this race‐specific resistance, as new pathogen races can rapidly evolve, rendering many resistance genes ineffective (Miller et al., 2020). This dynamic has been observed by many widely used *Pc* genes, which lose effectiveness as new pathogen races evolve. For example, virulent races have rendered *Pc36*, *Pc38*, *Pc39*, *Pc50*, *Pc68*, *Pc70*, and *Pc71* ineffective in certain regions (Moreau et al., 2024).

Recent studies have demonstrated the importance of partial or quantitative resistance as a more durable resistance to crown rust. Multiple minor‐effect genes largely control quantitative resistance and are often expressed as adult plant resistance (APR), which helps limit pathogen spread during the later stages of plant development (Díaz‐Lago et al., 2003; Leonard, 2002; Ohm & Shaner, 1992; Sunstrum et al., 2019; Winkler et al., 2016). The APR genes *Pc27*, *Pc28*, *Pc69*, *Pc72*, *Pc73*, and *Pc74* have only been identified in oat (Park et al., 2022). Unlike race‐specific resistance conferred by single major genes, which evolving pathogen populations can quickly overcome, additive resistance provides broad‐spectrum protection and reduces the risk of resistance breakdown. To enhance crown rust resistance in oat, breeders have increasingly focused on identifying quantitative trait loci (QTLs) associated with APR, a strategy that holds promise for durable disease resistance (Chowdhury et al., 2024; Lin et al., 2014; Nazareno et al., 2022; Rines et al., 2018).

These QTLs often exhibit additive effects, with lines homozygous for resistant alleles showing significantly lower disease severity (DS) (Lin et al., 2014; Nazareno et al., 2023). Integrating these QTLs and APR genes into breeding programs is critical for developing durable oat cultivars. Additive effects enhance resistance durability and effectiveness by enabling the synergistic contribution of multiple loci, leading to a more robust and stable defense that is less likely to be overcome by evolving pathogen populations (Nazareno et al., 2023). At the same time, this strategy reduces selection pressure on the pathogen, further supporting resistance breeding's long‐term potential. In addition to QTL mapping, gene mining has become valuable for identifying candidate genes associated with disease resistance. Analyzing genomic resources alongside disease‐resistance loci enables researchers to discover key metabolic pathways and biological processes contributing to plant defense mechanisms. This approach facilitates the identification of positional candidate genes, which can be further investigated to determine their functional roles in pathogen resistance.

These advances have laid the basis for meta‐QTL (MQTL) analysis, which provides an even more refined approach to improving crown rust resistance. Unlike conventional QTL mapping, which often relies on individual studies, MQTL analysis integrates data from multiple independent studies. This integration enhances the reliability of identifying QTLs by making them more stable across different genetic backgrounds and environmental conditions (Goffinet & Gerber, 2000; X. L. Wu & Hu, 2012). MQTL analysis also reduces the 95% confidence intervals (CIs) for QTLs, improving genomic positioning accuracy and increasing QTL utility in breeding programs (Martinez et al., 2016; Zhang et al., 2017). Narrowing the CI is a critical goal in genetic mapping, as it allows for more precise localization of the genes and underlying traits of interest (Kearsey & Farquhar, 1998). This increased accuracy allows for the development of molecular markers tightly linked to QTLs, thereby improving the efficiency of marker‐assisted selection (MAS) and enabling more targeted and effective breeding for crown rust resistance (Aloryi et al., 2022). Although MQTL analysis has been successfully applied to various crop species, such as wheat (S. Kumar et al., 2023; N. Pal et al., 2022; Saini et al., 2022; Vasistha et al., 2024), rice (Devanna et al., 2024; Goyal et al., 2024; I. S. Kumar & Nadarajah, 2020), and maize (M. Gupta et al., 2023; Sunitha et al., 2024), its application to oat has been limited by the complexity of its genome (Zhu & Kaeppler, 2003). These studies have demonstrated that MQTLs, also referred to as “MAS‐friendly MQTLs” (M. Gupta et al., 2023), provide more robust QTLs for MAS by addressing the problem of heterogeneity among QTL studies, thus refining QTL location and the magnitude of genetic effects (Saini et al., 2022). Such MAS‐friendly MQTLs can be effectively used for selection through associated molecular markers, enabling efficient selection for resistance traits and accelerating the development of improved oat cultivars. Progress has already been made in applying associated markers for disease resistance breeding in wheat (P. K. Gupta et al., 2022; S. Kaur et al., 2020; Sharma et al., 2021). This study represents the first MQTL mapping effort to elucidate the genetic architecture of oat crown rust resistance. This study will combine information from previously reported QTL with derived genomic data to identify candidate genes associated with resistance. Incorporating insights from oat genomics and regulatory elements associated with resistance mechanisms strengthens our understanding of the underlying genetic crown rust resistance pathway. Ultimately, these findings provide valuable tools for oat breeders and support the development of cultivars with durable crown rust resistance.

**Core Ideas**

- For genome‐wide meta‐analysis, 167 QTLs for crown rust resistance in oat were included.
- The study identified 23 meta quantitative trait loci (MQTL) regions, which provide the basis for breeding programs to improve oat resistance to crown rust.
- Gene mining within MQTL intervals revealed 1526 candidate genes associated with stress response and defense mechanisms.
- Functional analysis identified 12 key genes involved in plant defense, hormonal regulation, and stress adaptation.
- Findings provide valuable insights into developing oat cultivars with enhanced and durable crown rust resistance.

## 2 MATERIALS AND METHODS

### 2.1 Collection of data on QTL associated with crown rust resistance

**FIGURE 1:**
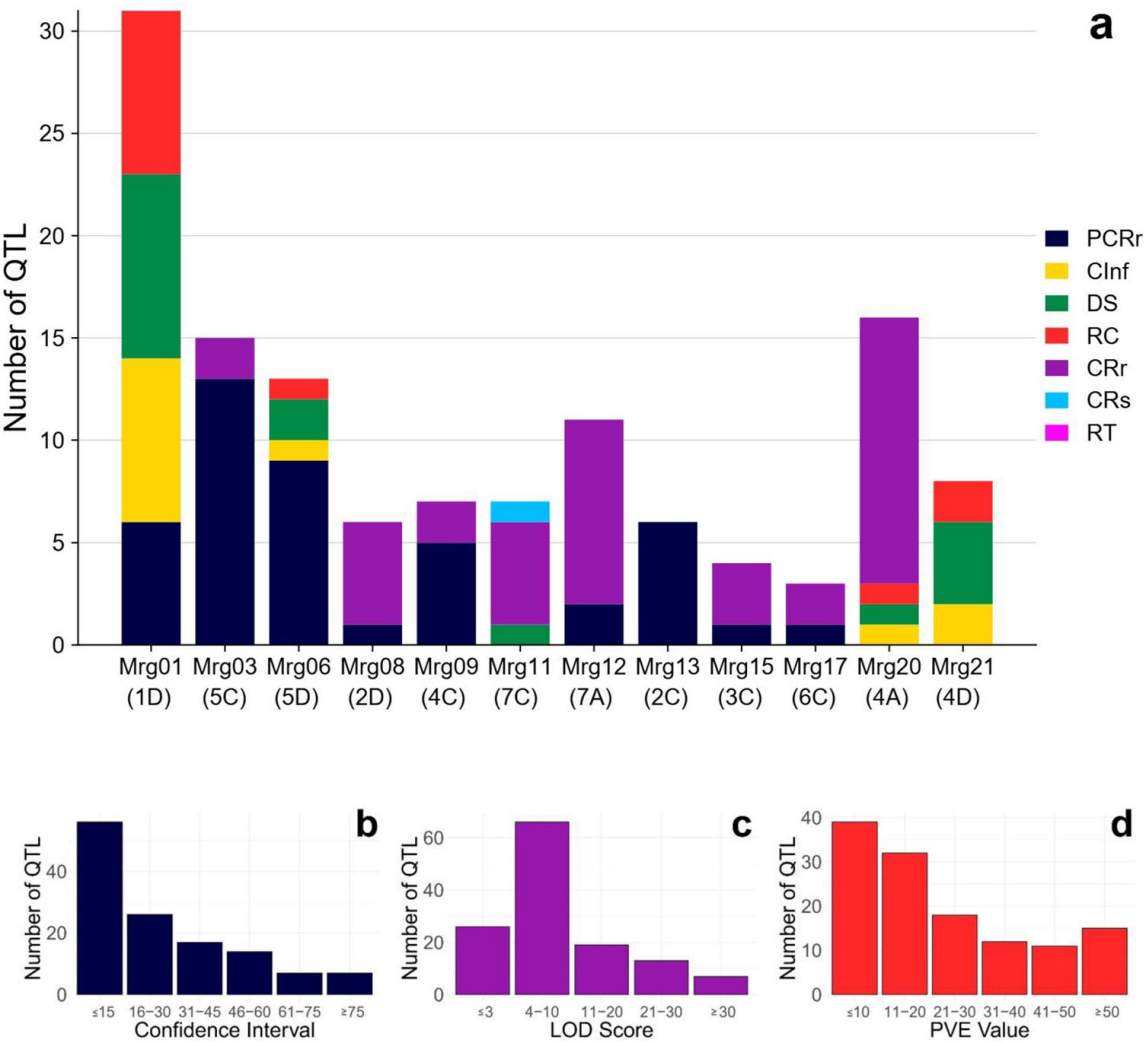
Quantitative trait locus (QTL) associated with crown rust resistance in oat were analyzed across chromosomes, focusing on (a) their chromosome‐wise distribution, (b) confidence intervals (CIs), (c) logarithm of odds (LOD) scores, and (d) phenotypic variation explained (PVE) values. Key traits studied include crown rust severity (CRs), reaction type (RT), partial crown rust resistance (PCRr), crown rust resistance (CRr), coefficient of infection (CInf), disease severity (DS), and reaction class (RC).

Research articles on crown rust resistance QTLs in the oat genome were systematically collected from reputable repositories and databases, such as Web of Knowledge, PubMed, Google Scholar, and other relevant, accessible data sources. The collected data consisted of detailed information on (i) the markers flanking the individual QTL, (ii) the peak positions and CIs of the identified QTL, (iii) the type and size of the mapping population used in the respective studies, and (iv) logarithm of odds (LOD) scores and phenotypic variation explained (PVE) or *R* ^2^ values (Table 1; Figure 1A–D). For cases where QTL peak positions were not explicitly reported in the source study, the midpoints of the two flanking markers were calculated and used as estimated peak positions. Similarly, when information on LOD scores was missing, a default threshold LOD score of 3.0 was applied to ensure uniformity in the analysis. During subsequent analyses, each QTL was assigned a unique identifier for clarity and consistency.

**TABLE 1:** Information about the oat (*Avena sativa*) populations used for the meta quantitative trait locus (MQTL) analysis.

Study	Population parents	Type of population	Population size	Marker system	Traits	Reference
1	Ogle (CI9401) × MAM17‐5	RIL	152	AFLP, RFLP, SSR	CRs, RT	Zhu and Kaeppler (2003)
2	MN841801‐1 × Noble‐2′	RIL	158	AFLP, RFLP, SCAR, SSR	PCRr, CRr	Portyanko et al. (2005)
3	UFRGS7 × UFRGS‐910906	F2	86	AFLP	PCRr	Barbosa et al. (2006)
		F6	90			
4	Ogle × TAM O‐301 (OT)	RIL	136	RFLP, AFLP, RAPD, STS, SSR	CRr	Jackson et al. (2008)
5	MN841801‐1 × Noble‐2′	RIL	150	AFLP, RFLP, SCAR, SSR	PCRr	Acevedo et al. (2010)
6	AC Assiniboia × MN841801	RIL	163	SNP	CInf, DS, RC	Lin et al. (2014)
	AC Medallion × MN841801	RIL	156			
	Makuru × MN841801	RIL	160			
7	Provena × CDC‐Boyer	RIL	148	SNP	CRr	Babiker et al. (2015)
	Provena × 94197A1‐9‐2‐2‐2‐5	RIL	145			
	CDC Boyer × 94197A1‐9‐2‐2‐2‐5	RIL	80			
8	TX07CS‐1948 × SA04213	RIL	178	SNP	CRr	Sunstrum et al. (2019)
9	Otana × CI9416‐2	RIL	130	SNP	CInf, DS, RC	Nazareno et al. (2022)
	Otana × PI189733	RIL	185			
10	OtanaA × CI1712‐5	RIL	191	SNP	CInf, DS, RC	Nazareno et al. (2023)
	OtanaA × CI17035‐1	RIL	172			
	OtanaI × PI263412	RIL	173			
11	PI 258731 × PI 573582	RIL	168	SNP	CInf, DS, RC	Chowdhury et al. (2024)

Abbreviations: AFLP, amplified fragment length polymorphism; CInf, coefficient of infection; CRr, crown rust resistance; CRs, crown rust severity; DS, disease severity; PCRr, partial crown rust resistance; RAPD, random amplified polymorphic DNA; RC, reaction class; RFLP, restriction fragment length polymorphism; RIL, recombinant inbred line; RT, reaction type; SCAR, sequence characterized amplified regions; SNP, single nucleotide polymorphism; SSR, simple sequence repeats; STS, sequence tagged sites.

The mapping study employed 19 mapping populations (comprising 17 recombinant inbred lines and one F_2_ population, and one F_6_ population), with population sizes ranging from 80 to 191 individuals evaluated across various environments and years (Table 1). These diverse populations provided a robust foundation for identifying QTL associated with crown rust resistance in oat. These linkage‐based mapping studies utilized a wide array of molecular markers to achieve high‐resolution genetic mapping, such as restriction fragment length polymorphism (RFLP), amplified fragment length polymorphism (AFLP), random amplified polymorphic DNA, sequence tagged sites, simple sequence repeats, sequence characterized amplified regions, and single nucleotide polymorphism markers. Using such a broad spectrum of marker systems reflects the evolution of genetic mapping technologies and their integration into oat research. These mapping studies identified 167 QTLs associated with crown rust resistance across various oat populations (Figure 1A). Among them, 62 QTLs were linked to partial crown rust resistance, 56 QTLs were linked to crown rust resistance, 18 QTLs were linked to DS, 12 QTLs were linked to coefficient of infection and reaction class, 4 QTLs were linked to CRs, and 3 QTLs were linked to reaction type. Of the 167 QTLs, 127 (76%) were successfully mapped onto the QTL consensus map. The remaining 40 QTLs could not be mapped due to the absence of common markers between the original linkage maps and the consensus map, which limited their alignment.

### 2.2 Evaluation of phenotypic data for crown rust QTL identification

Phenotypic data from 11 studies were analyzed to identify crown rust QTL, with most studies focusing on APR screening in the field, while some also included seedling resistance tests. Four studies conducted phenotyping at the Matt Moore Buckthorn Plots at the Minnesota Agricultural Experiment Station in Saint Paul, MN, where natural inoculation was facilitated using a buckthorn nursery (Babiker et al., 2015; Chowdhury et al., 2024; Nazareno et al., 2023, 2022). Five studies employed artificial field inoculations for APR phenotyping (Acevedo et al., 2010; Jackson et al., 2008; Lin et al., 2014; Portyanko et al., 2005; Zhu & Kaeppler, 2003), and one study used artificial field inoculations at another location (Chowdhury et al., 2024). Two studies conducted natural infection‐based evaluations of APR (Barbosa et al., 2006; Portyanko et al., 2005), and one study conducted additional evaluations under natural infection conditions (Babiker et al., 2015). One study only tested seedling resistance (Sunstrum et al., 2019), while another evaluated seedling resistance with two races in addition to APR testing (Chowdhury et al., 2024). These phenotyping approaches collectively provided a robust dataset for QTL analysis, encompassing diverse environmental conditions and experimental methodologies.

### 2.3 Initial QTL projection and MQTL prediction

**FIGURE 2:**
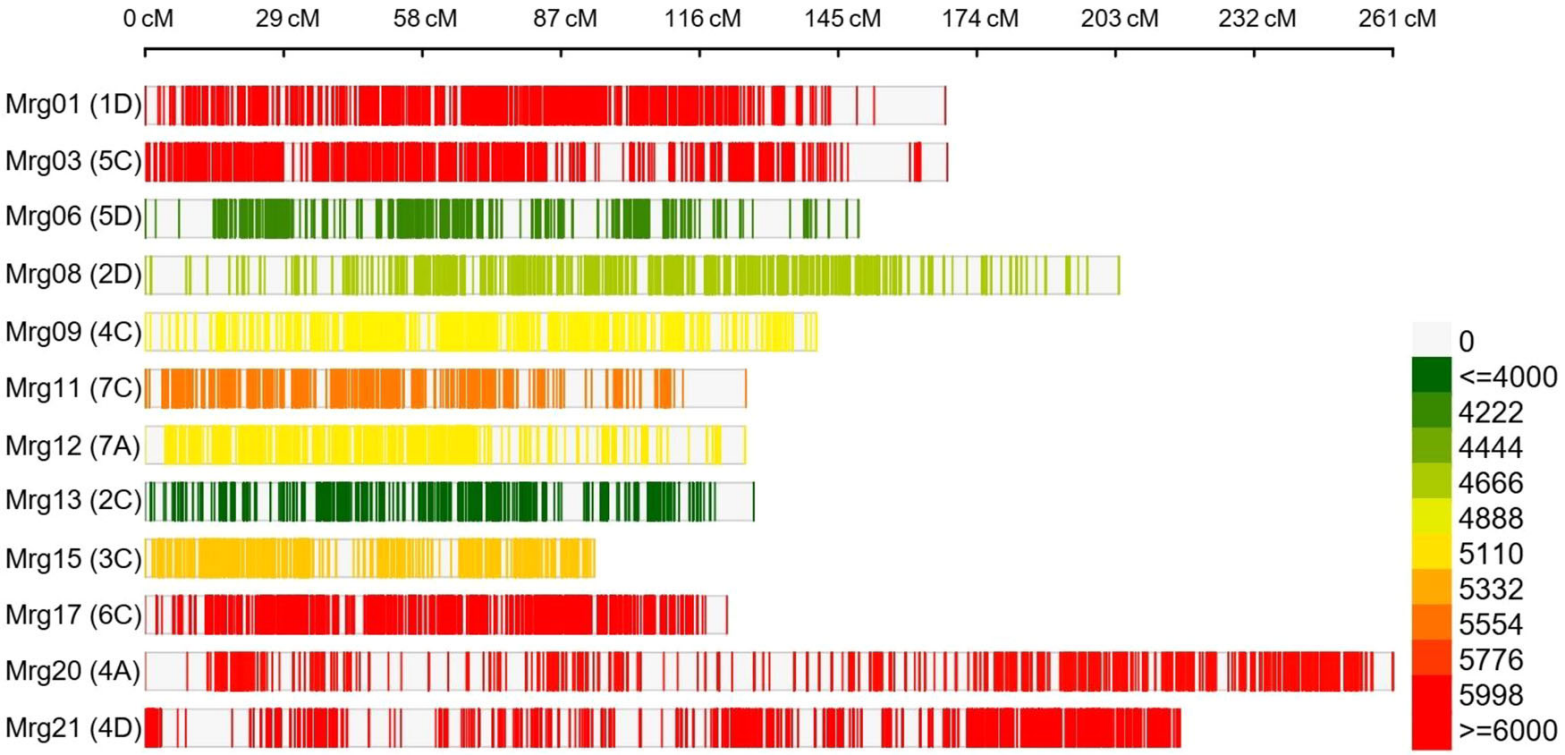
The distribution of the markers on the oat consensus map (OatConsensusMap‐2018; Bekele et al., 2018) for meta quantitative trait locus (MQTL) analysis in the present study (color intensity from green to red indicates low to high marker density, respectively).

After collecting the initial QTL data, all individual QTL were projected onto a reference map using BioMercator v4.2.3 software (Sosnowski et al., 2012). For this analysis, the high‐density genetic map (OatConsensusMap‐2018) was employed (Bekele et al., 2018). This map contains a dense set of 99,878 mapped molecular markers, spanning a total length of 2973.1 cM with a marker density of 33.6 markers per cM (Figure 2). QTLs that could not be aligned to the consensus map were excluded from further analysis. The linkage group nomenclature used in this study (e.g., Mrg01) follows the system established in the OatConsensusMap‐2018 (Bekele et al., 2018), while the alignment of these linkage groups with the AACCDD chromosome nomenclature was based on Jellen et al. (2024). This approach enables consistency with current standards in oat genetics research and facilitates reliable interpretation across studies. The projection of QTL onto the consensus map was performed using a homothetic function, assuming a linear relationship between the original QTL map and the reference map. This approach allowed for the estimation of most likely position of each QTL, along with its left and right flanking ends of the CI, based on common markers shared between QTL and reference maps (Chardon et al., 2004). The nearest common marker was used where flanking markers were unavailable, as long as they were located within a reasonable distance (≤10 cM) from the original peak. When no common markers were identified between the original QTL maps and the consensus map, an intermediary consensus map was used to bridge the original QTL maps to the OatConsensusMap‐2018 (Chaffin et al., 2016). This approach enabled reliable projection of QTLs onto the reference map, even without directly shared markers between the original and consensus maps. QTLs that could not be aligned due to missing shared markers, uncertain intervals, or other inconsistencies were excluded from further analysis.

The MQTL analysis was conducted using the Veyrieras two‐step algorithm (Veyrieras et al., 2007), which was applied individually to each chromosome. In the first step, the optimal QTL model was selected based on achieving the lowest criterion values in at least three of the five model selection criteria: Akaike information criterion (AIC), Corrected AIC, AIC Model‐3, Bayesian information criterion, and average weight of evidence criterion (Sosnowski et al., 2012). This approach ensured the selection of a robust and statistically supported model for MQTL analysis. In the second step, the selected model was utilized to determine the number of MQTL on each chromosome. The consensus locations of the MQTL were calculated using the variances of the initial QTL positions, while their 95% CIs were determined based on the variances of the QTL intervals (Sosnowski et al., 2012). The 23 MQTLs detected in this study were used to nominate positional candidate genes for gene mining analyses. For breeding implications, we selected MQTL based on the following stringent criteria: (i) a CI of less than 1 cM and (ii) the inclusion of at least five initial QTLs.

### 2.4 Determination of MQTL physical positions

Flanking marker positions on the PepsiCo OT3098 v2 hexaploid oat genome sequence were determined using tools in the T3/Oat (https://oat.triticeaetoolbox.org), GrainGenes (https://wheat.pw.usda.gov/GG3), and EnsemblPlants (https://plants.ensembl.org/index.html) databases, depending on marker type. The distribution and positions of MQTL within each linkage group were visualized as a heatmap using the RIdeogram R package (Hao et al., 2020), producing a visual graphical representation of their genomic locations.

### 2.5 Candidate genes and gene ontology (GO) enrichment analysis

Genes located in the MQTL intervals were identified through high‐confidence gene annotations from the oat reference genome sequence (Oat_OT3098_v2), available in the EnsemblPlants database (https://plants.ensembl.org/index.html). To gain functional insights into the identified genes, gene ontology (GO) enrichment analysis was conducted using the g:Profiler web tool (https://biit.cs.ut.ee/gprofiler/gost) (Kolberg et al., 2023). The analysis used an over‐representation model that assumes no GO term enrichment in the gene list (i.e., the null hypothesis). This model evaluates whether certain GO terms are over‐represented in the query gene list compared to a random sampling of terms associated with a positional candidate gene list of the same size. The *p*‐values for each GO term were adjusted for multiple testing using the g:SCS threshold method to control the false discovery rate. GO terms were considered significantly over‐represented compared to a random sampling of terms associated with a positional candidate gene list of the same size when the adjusted *p*‐value (Padj) was ≤0.05. This approach identified key biological processes, molecular functions, and cellular components associated with the candidate genes in the MQTL regions. Functional evidence for the GO domains of molecular function, cellular component, and biological processes was derived from the OT3098 reference genome, accessed through the EnsemblPlants database (https://plants.ensembl.org/index.html).

### 2.6 *Cis*‐acting regulatory element (CAREs) analysis

For the identification of *cis*‐acting regulatory element (CAREs), 1.5 kb upstream sequences from the 5′ untranslated regions of selected genes were extracted from the OT3098 v2 reference genome using the EnsemblPlants database (https://plants.ensembl.org/index.html). Candidate genes were selected by intersecting those associated with significantly enriched GO terms (identified via g:Profiler analysis) with genes located within the MQTL regions identified in this study. This filtering process resulted in a subset of 12 genes for further analysis. The upstream sequences of these genes were then analyzed using the PlantCARE database (http://bioinformatics.psb.ugent.be/webtools/plantcare/html/), which is a curated tool for identifying plant‐specific *cis*‐regulatory elements. The detected CAREs were annotated and categorized based on their known associations with biological functions such as hormone signaling, biotic stress responses, and transcription factor binding.

## 3 RESULTS

### 3.1 Crown rust resistance QTL and their distribution on the oat genome

The 127 mapped QTLs were distributed across 12 chromosomes with varying densities. The majority of QTLs were located on chromosomes 1D (31 QTLs), 4A (16 QTLs), 5C (15 QTLs), 5D (13 QTLs), and 7A (11 QTLs), while fewer QTLs were found on chromosomes 4D (8 QTLs), 4C (7 QTLs), 7C (7 QTLs), 2C (6 QTLs), 2D (6 QTLs), 3C (4 QTLs), and 6C (3 QTLs). The analysis revealed that crown rust resistance QTLs were predominantly distributed on the D sub‐genome (58/127, 45.7%) and the C sub‐genome (42/127, 33.1%), while fewer QTLs were mapped to the A sub‐genome (27/127, 21.3%) (Figure 1a, Table 2). Of the 127 mapped QTLs, 52 (40.9%) had CIs of less than 15 cM, whereas eight QTLs (6.3%) had CIs exceeding 75 cM (Figure 1b). The LOD scores of individual QTLs ranged from ≤3.0 to a maximum of 31.8. Among these, 42 QTLs had LOD scores between 4 and 10 (Figure 1c). The PVE by the QTL ranged from 2.9% to 75.8%, with an average PVE of 23.7%. Of the identified QTL, 38 exhibited PVE values of ≤10%, while 14 demonstrated PVE values exceeding 50% (Figure 1d).

**TABLE 2:** Identified meta quantitative trait locus (MQTL) associated with crown rust resistance in the oat (*Avena sativa*) genome (OatConsensusMap‐2018 and the Oat_OT3098_v2 reference genome).

MQTL	Chr. (genetic map)	Chr. (physical map)	Flanking markers	Position (cM)	CI (cM)	Physical position (Mb)	QTL No.	Number of genes within the MQTL interval
MQTL_(_ *_Pc_*_)_LG1.1	Mrg01	1D	GMI_ES03_c17272_213–GMI_ES02_c23703_243	2.8	1.01	483.01–485.33	9	22
MQTL_(_ *_Pc_*_)_LG1.2	Mrg01	1D	avgbs_74977.1.55–avgbs_103695.1.23	13.4	1.63	475.04–478.38	6	37
MQTL_(_ *_Pc_*_)_LG1.3	Mrg01	1D	avgbs_cluster_1998.1.48–avgbs_225027.1.53	73.62	0.06	352.58–359.17	16	128
MQTL_(_ *_Pc_*_)_LG3.1	Mrg03	5C	avgbs_213850.1.62–avgbs_115355.1.52	40.95	2.93	541.65–561.95	6	257
MQTL_(_ *_Pc_*_)_LG3.2	Mrg03	5C	avgbs2_99740.1.11–avgbs_90170.1.39	53.24	2.74	517.71–524.00	4	44
MQTL_(_ *_Pc_*_)_LG3.3	Mrg03	5C	avgbs_108983.1.64–GMI_GBS_9620	65.95	0.78	477.98–485.82	5	43
MQTL_(_ *_Pc_*_)_LG6.1	Mrg06	5D	avgbs_111562.1.63–avgbs_49594.1.48	38.22	2.81	464.40–471.65	4	60
MQTL_(_ *_Pc_*_)_LG6.2	Mrg06	5D	avgbs_221341.1.39–avgbs_cluster_23665.1.10	96.82	0.36	41.51–57.75	9	125
MQTL_(_ *_Pc_*_)_LG8.1	Mrg08	2D	avgbs_cluster_28306.1.46–GMI_DS_oPt‐17694_374	25.58	0.02	5.50–9.44	6	34
MQTL_(_ *_Pc_*_)_LG9.1	Mrg09	4C	GMI_ES02_lrc37077_735–avgbs_41527.1.45	3.67	1.78	5.57–5.74	3	5
MQTL_(_ *_Pc_*_)_LG9.2	Mrg09	4C	avgbs_cluster_30129.1.15–avgbs_cluster_25913.1.32	111.5	4.06	528.07–528.82	4	7
MQTL_(_ *_Pc_*_)_LG11.1	Mrg11	7C	avgbs_205234.1.49–avgbs_117385.1.38	19.53	1.24	645.41–651.26	7	0
MQTL_(_ *_Pc_*_)_LG12.1	Mrg12	7A	UMN295A–avgbs_cluster_6825.1.9	0.64	0.27	0.37–0.54	5	4
MQTL_(_ *_Pc_*_)_LG12.2	Mrg12	7A	avgbs_96007.1.25–avgbs_cluster_26522.1.17	11.89	0.02	483.42–491.73	6	70
MQTL_(_ *_Pc_*_)_LG13.1	Mrg13	2C	avgbs_cluster_5234.1.18–avgbs2_164428.1.63	12.93	0.26	1.64–8.79	6	81
MQTL_(_ *_Pc_*_)_LG15.1	Mrg15	3C	avgbs_115950.1.42–avgbs_cluster_44255.1.62	58.64	0.54	19.71–23.12	4	29
MQTL_(_ *_Pc_*_)_LG17.1	Mrg17	6C	GMI_ES17_c17442_334–avgbs2_200054.1.18	8.05	2.12	0.32–1.49	3	11
MQTL_(_ *_Pc_*_)_LG20.1	Mrg20	4A	CSU25–UMN363B	2.87	0.58	0.53–0.54	3	1
MQTL_(_ *_Pc_*_)_LG20.2	Mrg20	4A	GMI_ES15_c7632_384–avgbs_11193.1.11	17.3	0.58	178.05–194.81	3	89
MQTL_(_ *_Pc_*_)_LG20.3	Mrg20	4A	avgbs_109050.1.51–GMI_ES05_c2066_503	28.05	0.71	228.87–229.78	2	7
MQTL_(_ *_Pc_*_)_LG20.4	Mrg20	4A	GMI_ES22_c4463_275–avgbs_16201.1.59	33.22	0.45	229.78–236.56	5	44
MQTL_(_ *_Pc_*_)_LG20.5	Mrg20	4A	avgbs_cluster_32781.1.30–avgbs_120857.1.15	72.4	5.69	252.01–268.42	3	192
MQTL_(_ *_Pc_*_)_LG21.1	Mrg21	4D	avgbs2_30300.1.56–avgbs_cluster_23336.1.30	96.3	0.04	236.20–261.93	8	236

Abbreviations: CI, confidence interval; Chr., chromosome.

### 3.2 Consensus map and MQTL for crown rust resistance

**FIGURE 3:**
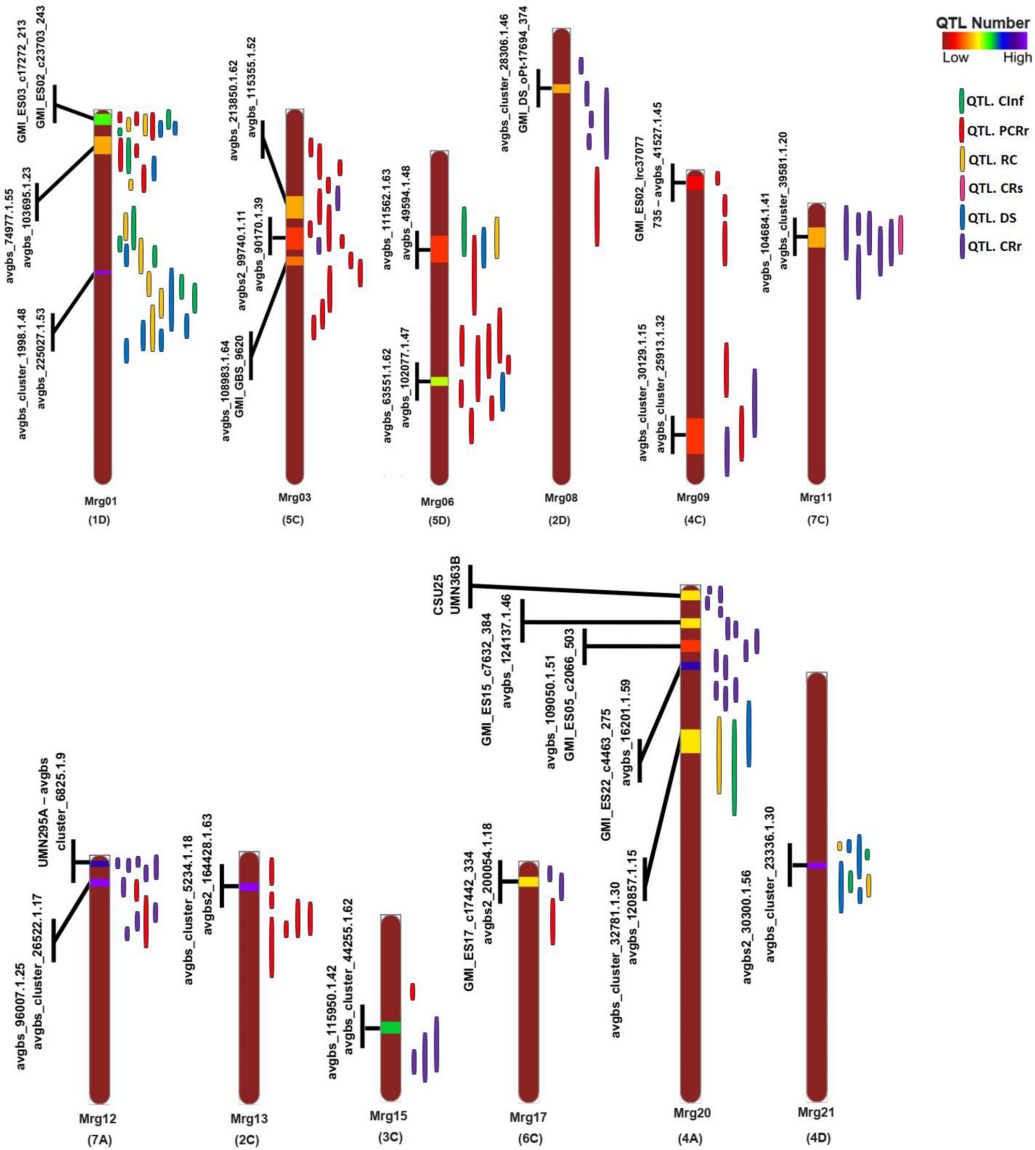
Position of detected meta quantitative trait locus (MQTL) on the oat genome (OatConsensusMap‐2018; Bekele et al., 2018) associated with crown rust resistance with a 95% confidence interval. Each color in a different linkage group indicates the number of initial quantitative trait locus (QTL) involved in each MQTL. The flanking markers for each MQTL are presented on the left side of the linkage groups.CInf, coefficient of infection; CRr, CRs, crown rust resistance; crown rust severity; DS, disease severity; PCRr, partial crown rust resistance; RC, reaction class.

Twenty‐three MQTLs were identified for crown rust resistance based on 127 mapped QTLs on the consensus map (Figure 3; Table 2). Among the sub‐genomes, seven MQTLs were determined on sub‐genome A, with chromosome 4A containing the highest number (five MQTLs) and chromosome 7A containing two MQTLs. Similarly, seven MQTLs were predicted on sub‐genome D, with chromosome 1D harboring the maximum (three MQTLs), followed by two MQTLs on chromosome 5D, while chromosomes 2D and 4D each displayed a single MQTL. Sub‐genome C had the highest number of MQTL, with nine in total. Chromosome 5C harbored the maximum (three MQTLs) of these, followed by two MQTLs on chromosome 4C and one MQTL each on chromosomes 2C, 3C, 6C, and 7C (Figure 3; Table 2).

The number of QTL contributing to individual MQTL varied significantly, ranging from ≤3 QTL in 6 MQTLs to ≥6 QTLs in 10 MQTLs, including MQTL_(_ *_Pc_*_)_1D.1, MQTL_(_ *_Pc_*_)_1D.2, MQTL_(_ *_Pc_*_)_1D.3, MQTL_(_ *_Pc_*_)_5C.1, MQTL_(_ *_Pc_*_)_5D.2: MQTL_(_ *_Pc_*_)_2D.1, MQTL_(_ *_Pc_*_)_7C.1, MQTL_(_ *_Pc_*_)_7A.2, MQTL_(_ *_Pc_*_)_2C.1, and MQTL_(_ *_Pc_*_)_4D.1 (Figure 3, Table 2). The CIs of the reported MQTL ranged from 0.02 to 5.69 cM, with an average of 1.37 cM, representing a 5.55‐fold reduction compared to the CIs of the original QTL (Table 2). All 23 MQTLs were physically anchored to the oat reference genome. The physical CIs of the MQTL ranged from 0.01 (MQTL_(_ *_Pc_*_)_4A.1) to 25.73 Mb (MQTL_(_ *_Pc_*_)_4D.1), with a mean physical CI of 7.29 Mb (Table 2).

### 3.3 Gene mining and GO analysis within MQTL regions

**FIGURE 4:**
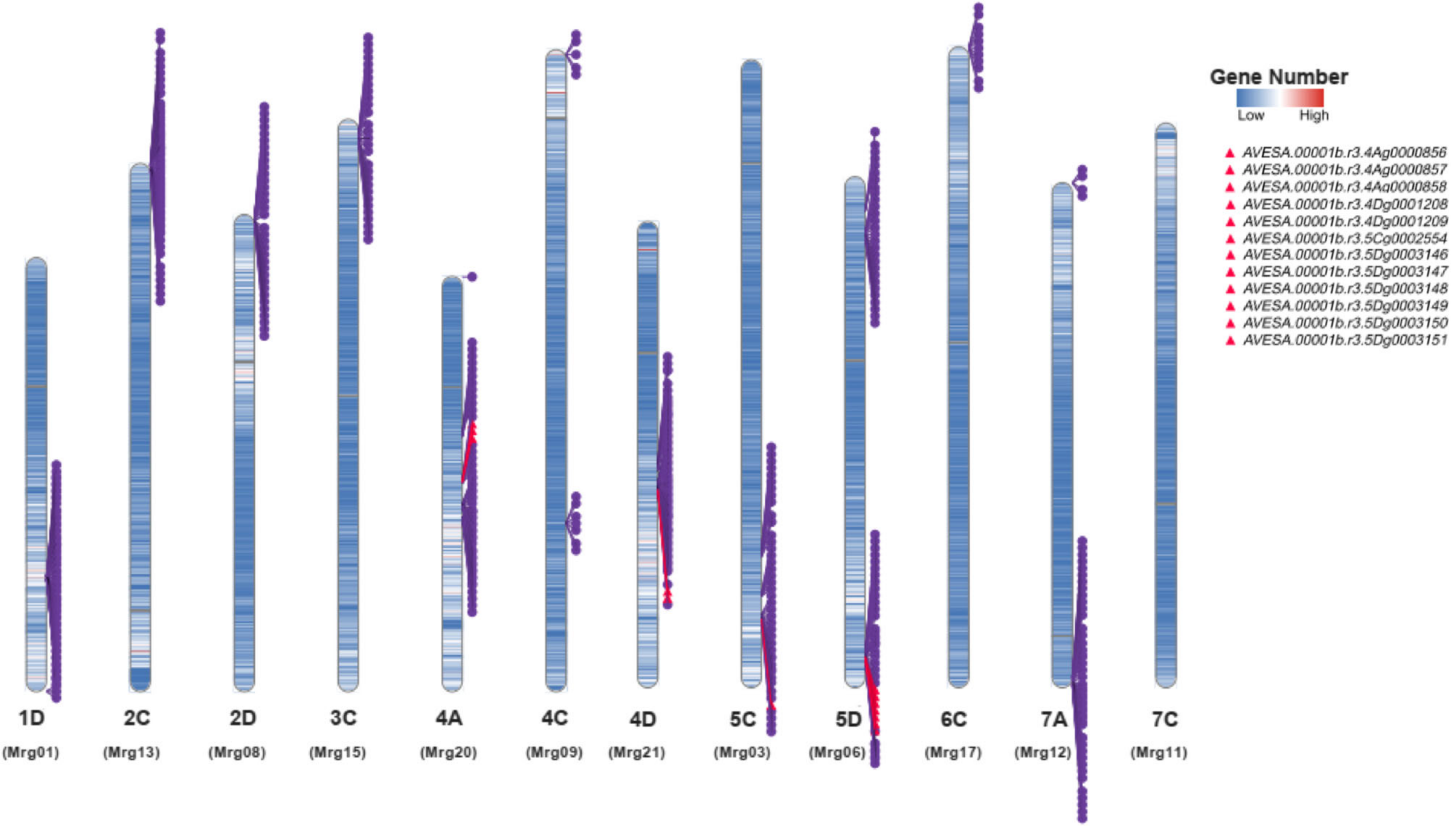
A heat map illustrates the gene density of *Avena sativa* chromosomes (Oat_OT3098_v2). The positions of the genes detected at each meta quantitative trait locus (MQTL) interval are displayed on the right side of the chromosomes. Pink triangles indicate significant functional candidate genes associated with crown rust resistance.

Table 2 presents the number of candidate genes identified in the intervals of the detected MQTL, while detailed annotations for all genes in each MQTL interval are available in Table S1. Gene mining in crown rust resistance MQTL revealed 1526 unique genes. Collectively, the MQTL detected on the chromosomes in the D sub‐genome contained the largest number of genes (642), followed by the C sub‐genome with 477 genes and the A sub‐genome with 407 genes (Figure 4; Table 2; Table S1). The MQTL with the highest number of candidate genes was MQTL_(_ *_Pc_*_)_5C.1, containing 257 genes, followed by MQTL_(_ *_Pc_*_)_4D.1 with 236 genes (Table 2). Interestingly, the number of predicted genes largely corresponds to the size of the MQTL 95% CIs, with larger MQTL CIs encompassing more genes. On the other hand, MQTL_(_ *_Pc_*_)_4A.1, which had the smallest size, contained only one gene, while MQTL_(_ *_Pc_*_)_7A.1 and MQTL_(_ *_Pc_*_)_4C.1 contained four and five genes, respectively (Figure 4; Table 2).

**FIGURE 5:**
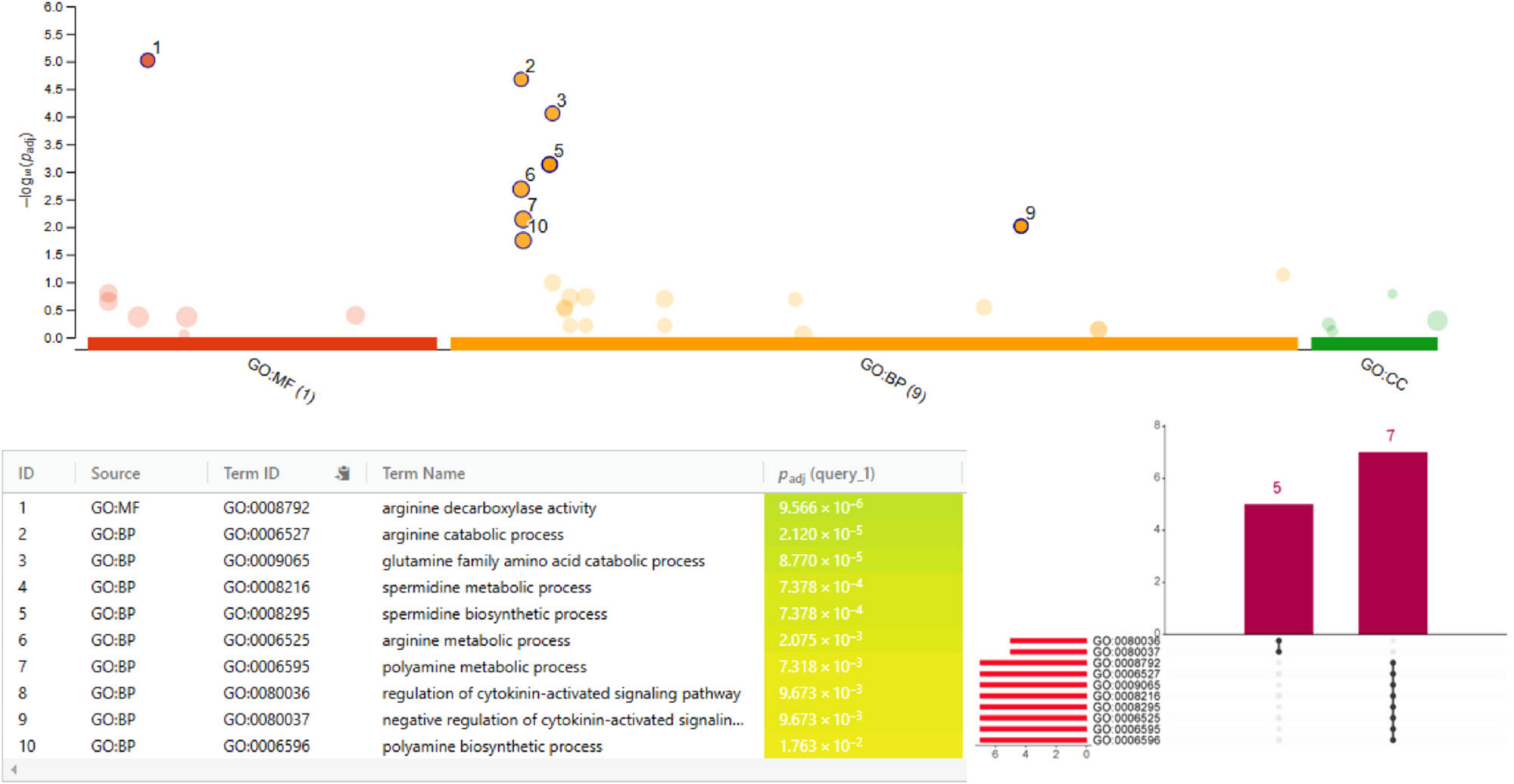
Gene ontology (GO) and functional enrichment analysis of candidate genes in meta quantitative trait locus (MQTL) regions of the oat genome (Oat_OT3098_v2) in response to crown rust revealed important insights into their functional roles. Each circle represents a significant GO term, with significance determined by an adjusted *p*‐value (*p*_adj_ ≤ 0.05) using G:Profiler. The *y*‐axis represents −log10(*p*_adj_), and circles are color‐coded to indicate their respective GO domains: molecular function (MF), cellular component (CC), and biological process (BP), highlighting the diverse roles of the identified genes in crown rust resistance.

Among the candidate genes identified within the MQTL regions, 12 genes were determined for their potential significance based on functional annotations, which were arginine decarboxylase (ADC) activity, glutamine metabolism, spermidine biosynthesis, polyamine metabolism, and the regulation of cytokinin‐activated signaling pathways. These include *AVESA.00001b.r3.5Dg0003146*, *AVESA.00001b.r3.5Dg0003147*, *AVESA.00001b.r3.5Dg0003148*, *AVESA.00001b.r3.5Dg0003149*, *AVESA.00001b.r3.5Dg0003150*, *AVESA.00001b.r3.5Dg0003151*, *AVESA.00001b.r3.5Cg0002554*, *AVESA.00001b.r3.4Ag0000856*, *AVESA.00001b.r3.4Ag0000857*, *AVESA.00001b.r3.4Ag0000858*, *AVESA.00001b.r3.4Dg0001208*, and *AVESA.00001b.r3.4Dg0001209* (Table 3). GO analysis revealed diverse and significant functions associated with these genes, including (i) ADC activity and arginine metabolic processes, (ii) glutamine family amino acid catabolic processes, (iii) spermidine metabolism and biosynthesis, (iv) polyamine metabolism and biosynthesis, and (v) the regulation of cytokinin‐activated signaling pathways (Figure 5).

**TABLE 3:** The most prevalent *cis*‐regulatory elements in the promoter of crown rust resistance responsive genes in oat.

*Cis*‐regulatory element	Function	*Cis*‐regulatory element	Function
A‐box	*Cis*‐acting regulatory element	HD‐Zip 1	Element involved in differentiation of the palisade mesophyll cells
AAAC‐motif	Light responsive element	I‐box	Part of a light responsive element
AAGAA‐motif	–	LTR	*Cis*‐acting element involved in low‐temperature responsiveness
ABRE	*Cis*‐acting element involved in the abscisic acid responsiveness	MBS	MYB binding site involved in drought‐inducibility
ABRE2		MBSI	MYB binding site involved in flavonoid biosynthetic genes regulation
ABRE3a	–	MRE	MYB binding site involved in light responsiveness
ABRE4	–	MYB	–
AC‐I	–	MYB recognition site	–
ACTCATCCT sequence	–	MYB‐like sequence	–
AE‐box	Part of a module for light response	MYC	–
ARE	*Cis*‐acting regulatory element essential for the anaerobic induction	Myb	–
AT‐rich element	Binding site of AT‐rich DNA binding protein (ATBP‐1)	Myb‐binding site	–
ATCT‐motif	Part of a conserved DNA module involved in light responsiveness	Myc	–
AT1‐motif	Part of a light responsive module	NON‐box	–
AT∼TATA‐box	–	O2‐site	*Cis*‐acting regulatory element involved in zein metabolism regulation
Box 4	Part of a conserved DNA module involved in light responsiveness	RY‐element	Cis‐acting regulatory element involved in seed‐specific regulation
Box II	Part of a light responsive element	P‐box	Gibberellin‐responsive element
Box II ‐like sequence	*Cis*‐acting regulatory element	STRE	–
CAAT‐box	Common *cis*‐acting element in promoter and enhancer regions	Sp1	Light responsive element
CAT‐box	*Cis*‐acting regulatory element related to meristem expression	TATA	–
CCAAT‐box	Mybhv1 binding site	TATA‐box	Core promoter element around ‐30 of transcription start
CCGTCC motif	–	TATC‐box	Cis‐acting element involved in gibberellin‐responsiveness
CGTCA‐motif	*Cis*‐acting regulatory element involved in the MeJA‐responsiveness	TC‐rich repeats	*Cis*‐acting element involved in defense and stress responsiveness
CTAG‐motif	–	TCA	–
DRE core	–	TCA‐element	*Cis*‐acting element involved in salicylic acid responsiveness
ERE	–	TCCC‐motif	Part of a light responsive element
DRE1	–	TCT‐motif	Part of a light responsive element
G‐Box	*Cis*‐acting regulatory element involved in light responsiveness	TGA‐element	Auxin‐responsive element
G‐box	*Cis*‐acting regulatory element involved in light responsiveness	TGACG‐motif	*Cis*‐acting regulatory element involved in the MeJA‐responsiveness
GA‐motif	Part of a light responsive element	W box	–
GATA‐motif	Part of a light responsive element	WRE3	–
GC‐motif	Enhancer‐like element involved in anoxic specific inducibility	WUN‐motif	–
GCN4_motif	*Cis*‐regulatory element involved in endosperm expression	as‐1	–
GT1‐motif	Light responsive element	box S	–
		circadian	*Cis*‐acting regulatory element involved in circadian control

Abbreviation: MeJA, methyl jasmonate.

### 3.4 Identification of CAREs in the promoter site of functional genes

**FIGURE 6:**
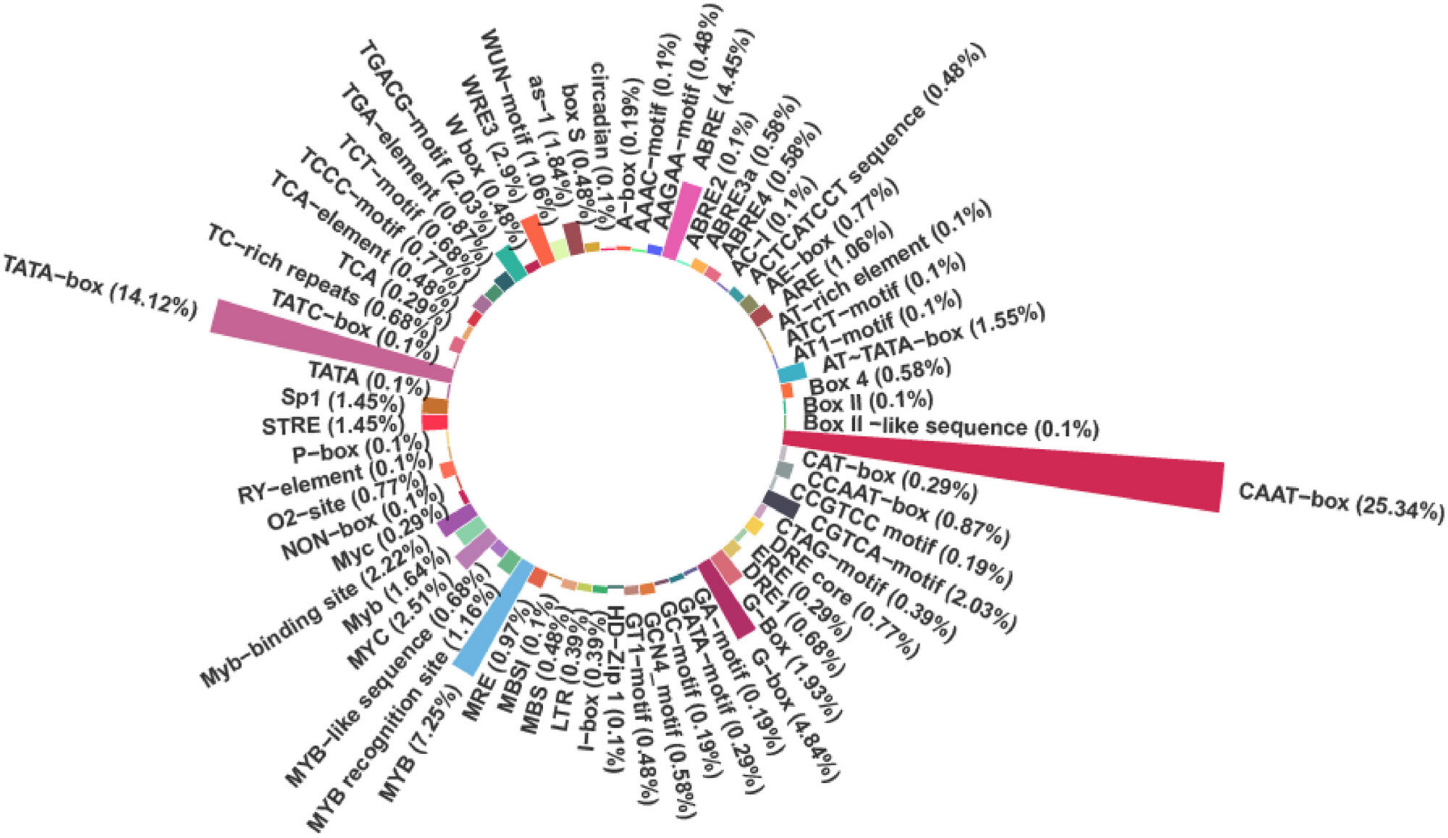
Distribution of major *cis*‐acting regulatory elements at the oat's promoter site of crown rust resistance genes.

The identified CAREs in the promoter regions of the 12 genes located within the crown rust resistance MQTL regions in oat were presented in Figure 6 and Table 3. These CAREs were predominantly linked to functions related to light response, hormonal regulation, and stress response, which are critical for the regulation of crown rust resistance genes in oat (Table 3). Key elements identified included the CAAT‐box (25.34%) and the TATA‐box (14.12%), which are essential sequences in the promoter regions of many genes and play crucial roles in transcriptional regulation. Hormonal regulatory elements identified in the promoter regions included those responsive to methyl jasmonate (MeJA), gibberellic acid (GA), and abscisic acid (ABA), such as ABA responsive element (ABRE; 4.45%), CGTCA‐motif (2.03%), TGACG‐motif (2.03%), P‐box (0.1%), and TATC‐box (0.1%) (Table 3). Light‐responsive CAREs were also abundant in the promoter regions of the detected resistance genes. These included G‐box (4.84%), G‐Box (1.93%), TCCC‐motif (0.77%), TCT‐motif (0.68%), GT1‐motif (0.48%), I‐box (0.39%), GATA‐motif (0.29%), GA‐motif (0.19%), AAAC‐motif (0.1%), and ATCT‐motif (0.1%) (Table 3). These elements highlight the role of light‐regulated transcriptional processes in crown rust resistance gene expression. Aside from this, stress‐responsive elements were present in the promoter regions, such as MYB (7.25%), MYC (2.51%), TC‐rich repeats (0.68%), and the circadian element (0.1%) (Table 3).

## 4 DISCUSSION

This study provides valuable insights into the genetic architecture of crown rust resistance in oat, achieved through the comprehensive dissection of QTL and MQTL associated with this globally significant trait. Identifying key candidate genes and refined genomic regions contributing to crown rust resistance in *Avena* species has substantial relevance for resistance breeding and the sustainable management of crown rust, which remains a major constraint to oat production worldwide. The MQTL analysis conducted in this study consolidated 127 QTLs into 23 robust MQTLs across 12 oat chromosomes, significantly improving the precision and stability for mapping crown rust resistance. Chromosomes 4A, 1D, and 5C contained the highest number of MQTL, with five, three, and three MQTLs, respectively, indicating their critical roles in harboring resistance loci. Among the detected MQTL, MQTL_(_ *_Pc_*_)_1D.3, MQTL_(_ *_Pc_*_)_1D.1, MQTL_(_ *_Pc_*_)_5D.2, and MQTL_(_ *_Pc_*_)_4D.1 emerged as the most promising candidates for breeding implications, as they exhibited the highest number of initial QTL identified across independent populations. These MQTLs represent viable, stable, and robust loci effective under diverse experimental conditions, making them key targets for further genetic exploration. The stability of these MQTLs across different populations and conditions reinforces their utility in breeding programs to improve resistance, durability, and adaptability. The improved resolution achieved through this MQTL analysis enhances our understanding of the genomic landscape associated with crown rust resistance. With this approach, CIs have been reduced and data from multiple studies have been incorporated, thereby increasing the accuracy of mapping resistance loci and enabling the identification of candidate genes. This will facilitate future fine‐mapping efforts, the development of high‐resolution markers, and the functional validation of resistance‐associated genes (Jackson et al., 2008; Löffler et al., 2009). In this study, MQTL analysis was conducted using QTL for crown rust resistance previously reported in independent experiments. This approach aimed to enhance our understanding of genetic regulation of crown rust resistance in oat. The process began with projecting the original QTL onto a consensus map, a critical first step in identifying consensus regions through meta‐analysis. This projection refines QTL positions, reducing their CIs and increasing their reliability for breeding applications.

The genetics of quantitative resistance to crown rust has been extensively studied in various oat species using QTL mapping methodologies (Acevedo et al., 2010; Barbosa et al., 2006; Chowdhury et al., 2024; Jackson et al., 2008; Lin et al., 2014; Nazareno et al., 2018; Portyanko et al., 2005; Sunstrum et al., 2019; Zhu & Kaeppler, 2003). These studies have identified numerous QTLs associated with crown rust resistance in oat. However, QTL identified in one mapped population often fail to perform well in breeding programs involving different populations or parental lines (S. Kumar et al., 2023; Yang et al., 2021). This reflects the inherent challenges in dissecting the genetic architecture of complex traits, which remains a significant obstacle for plant breeders and geneticists. While traditional QTL mapping approaches, such as linkage mapping, have been instrumental in identifying marker‐trait associations for complex traits like partial resistance to crown rust, their limitations are well‐documented. These include confounding effects from genetic background, environmental variation, limited marker density, and low mapping resolution. MQTL analysis addresses these challenges by integrating QTL from multiple studies, refining their positions, and identifying those that are stable and robust across diverse genetic and environmental contexts (S. Kaur et al., 2023; Pascual et al., 2016; Y. Xu et al., 2017). Unlike traditional methods, MQTL analysis reduces heterogeneity between studies and narrows CIs, improving the utility of QTL in breeding programs (Arcade et al., 2004; S. Kaur et al., 2023; S. Kumar et al., 2023; Saini et al., 2022).

The distribution of crown rust resistance genes, categorized into seedling resistance and APR genes, was analyzed across seven *Avena* species using previously published data (Park et al., 2022). The MQTL and candidate genes identified in this study provide new insights into the genetic architecture of crown rust resistance in oat. In *A. sativa*, 13 seedling resistance genes and two APR genes were previously reported (Park et al., 2022), and our analysis identified 22 MQTLs linked to crown rust resistance in this species, encompassing key genomic regions associated with durable resistance. Eleven of these MQTLs contain nine candidate genes associated with APR, which demonstrates their potential importance in developing improved resistance in breeding programs (Figure 4). *Avena strigosa* has been recognized as an important source of resistance, with 22 resistance genes identified (Park et al., 2022), and our study detected one MQTL in this species that harbors three functional resistance genes linked to crown rust resistance (Figure 4). Although *A. byzantina* has 13 *R* genes and *A. sterilis* contains 41 seedling resistance genes and four APR genes (Park et al., 2022), but no MQTLs were detected in these species. Other species, such as *Avena glabrata*, *Avena trichophylla*, and *Avena longiglumis*, exhibit limited resistance sources, with only one or two *R* genes and no reported APR genes (Park et al., 2022). Accordingly, no MQTLs were identified in these species, underscoring their minimal contribution to crown rust resistance.

### 4.1 Candidate gene mining in the MQTL and their association with crown rust resistance

Candidate gene mining from the 23 identified MQTLs revealed several genes linked to key biological processes, providing insights into crown rust resistance mechanisms. Polyamines (PAs) play a crucial role in plant‐pathogen interactions by amplifying pattern‐triggered immunity through the production of reactive oxygen species (ROS) (Gerlin et al., 2021). Among the identified candidate genes, *AVESA.00001b.r3.5Dg0003146*, *AVESA.00001b.r3.5Dg0003147*, *AVESA.00001b.r3.5Dg0003148*, *AVESA.00001b.r3.5Dg0003149*, *AVESA.00001b.r3.5Dg0003150*, and *AVESA.00001b.r3.5Dg0003151* were closely linked to polyamine metabolism, namely, ADC activity and spermidine biosynthesis. As part of the metabolism of PAs, ADC converts arginine to agmatine, which serves as a precursor for PAs such as putrescine, spermidine, and spermine (Blázquez, 2024; Gerlin et al., 2021; M. Pal & Janda, 2017; Zeier, 2013). These PAs enhance plant defense by strengthening cell walls, scavenging ROS, and mitigating oxidative damage during pathogen infection. In oat, increased ADC activity has been associated with improved resistance to *Pca*, particularly during the critical pre‐penetration and penetration stages of infection (Montilla‐Bascón et al., 2016; Winter et al., 2015; Zeier, 2013). These findings demonstrate the importance of polyamine metabolism in increasing plant defenses against crown rust.

Amino acids like arginine and glutamine play dual roles as essential protein building blocks and regulators of stress responses (J. Cai & Aharoni, 2022; Hildebrandt et al., 2015). The gene *AVESA.00001b.r3.5Cg0002554*, associated with arginine metabolism, underscores the importance of arginine as a precursor for polyamine synthesis, which supports plant development and enhances resilience to stress (Bagni & Tassoni, 2001; Winter et al., 2015). Similarly, glutamine is critical for nitrogen metabolism, incorporating ammonium into key metabolic pathways essential for plant growth and stress tolerance. Overexpression of glutamine synthetase genes has been shown to improve drought and salt tolerance by maintaining photosystem function, mitigating oxidative stress, and supporting photorespiration (Ganie, 2021; Hoshida et al., 2000; James et al., 2018; Y. Liu et al., 2017). The genes linked to glutamine metabolism identified in this study further emphasize their role in enhancing plant defenses against biotic stress. This is achieved through regulating ROS levels and enhancing antioxidant capacity, highlighting glutamine's importance in strengthening plant immunity.

Cytokinin signaling balances plant growth and defense, enabling plants to allocate resources effectively based on environmental conditions (Akhtar et al., 2020; Pieterse et al., 2014). In this study, several identified genes, including *AVESA.00001b.r3.4Ag0000856*, *AVESA.00001b.r3.4Ag0000857*, *AVESA.00001b.r3.4Ag0000858*, *AVESA.00001b.r3.4Dg0001208*, and *AVESA.00001b.r3.4Dg0001209*, were associated with the regulation and negative regulation of cytokinin‐activated signaling pathways. Cytokinin influences various processes related to plant development and pathogen resistance (Kieber & Schaller, 2018). It also interacts with other hormonal pathways, such as jasmonic acid (JA), to enhance plant defense mechanisms (Akhtar et al., 2020; Prasad, 2022). These findings underline cytokinin's critical role in modulating nutrient responses and strengthening resistance to pathogens, particularly crown rust (Cortleven et al., 2019). The identification of candidate genes involved in cytokinin signaling suggests their potential role in enhancing resistance mechanisms. The study also demonstrated the importance of polyamine metabolism, amino acid pathways, and cytokinin signaling in crown rust resistance, revealing diverse biochemical and regulatory pathways that enhance plant defenses. Functional annotations of these candidate genes demonstrate their potential to strengthen resistance mechanisms, making them valuable targets for further validation. These insights can be utilized in MAS strategies for integrating these genes into breeding programs. This approach may enhance the resilience of oat cultivars to crown rust, ensuring sustainable production in biotic stress conditions.

### 4.2 Functional implications of *cis*‐acting regulatory elements in oat crown rust resistance

Identifying CAREs in the promoter regions of functional genes associated with crown rust resistance in oat reveals their significant role in transcriptional regulation and plant defense (Cui et al., 2023). The promoter types selected for this analysis were chosen based on two primary criteria: (i) their established relevance to biotic stress responses, particularly in relation to crown rust resistance, and (ii) their frequency within the promoter regions of the 12 candidate genes under investigation. Elements such as ABRE, MYB, MYC, TGACG‐motif, and TC‐rich repeats have been documented in previous reports for their functional association with plant defense mechanisms, hormonal signaling, and stress adaptation (Hou et al., 2016; Huda et al., 2013; Li et al., 2020). Transcriptional regulation involves the interaction between transcription factors and specific CAREs in the promoter regions, making these elements essential for orchestrating plant immune responses (Kaur & Pati, 2016; A. Kaur et al., 2017). CAREs are key regulatory units in plant genomes that control gene expression in response to biotic and abiotic stresses, as highlighted by their role in regulating resistance mechanisms to crown rust in oat (Cui et al., 2023; A. Kaur et al., 2017). The CAREs identified in this study were primarily associated with hormonal regulation, light response, and stress response, emphasizing the complexity of the plant immune system. These categories demonstrate how diverse regulatory elements collaborate to enable resistance against pathogens like crown rust.

Hormonal regulation is central in shaping plant defense mechanisms against biotic stresses. This study identified several hormone‐related CAREs, particularly ABRE, CGTCA‐motif, TGACG‐motif, P‐box, and TATC‐box, which are involved in the regulation of hormones such as ABA and JA (Shariatipour & Heidari, 2018; Shariatipour & Heidari, 2020). ABA, a key phytohormone, mediates plant responses to stress by regulating defense pathways. Its role in resistance to both abiotic and biotic stresses is well‐documented, with the ABRE element acting as a crucial mediator in ABA‐induced gene expression (Hewage et al., 2020; Rai et al., 2024). Jasmonates, including JA and MeJA, are lipid‐derived hormones that regulate defense responses under stress. The identified TGACG and CGTCA motifs, associated with MeJA response, are critical for activating jasmonate signaling pathways, which help plants combat pathogen attacks (Rehman et al., 2023; Siddiqi & Husen, 2019). GA is also involved in plant growth and defense regulation. The presence of GA‐related CAREs in crown rust resistance genes suggests that GA signaling modulates immune responses, further illustrating the interconnected nature of plant hormone pathways (Hedden, 2020; K. Wu et al., 2021; H. Xu et al., 2014). These elements suggest hormonal signaling pathways actively modulate crown rust resistance in oat.

Light‐responsive CAREs such as G‐box, TCCC‐motif, GT1‐motif, and GATA‐motif were identified in the promoter regions of crown rust resistance genes. These elements regulate gene expression in response to light, influencing plant growth, development, and immune responses. As a critical environmental factor, light modulates defense mechanisms by coordinating immune‐related pathway activation (A. Kaur et al., 2017). The presence of light‐responsive motifs highlights their role in enhancing plant adaptability to environmental conditions that may affect pathogen growth and infection. Stress‐responsive CAREs, like MYB, MYC, TC‐rich repeats, and TCA elements are crucial in regulating plant responses to biotic stress. MYB transcription factors regulate secondary metabolites like phenolics and flavonoids, which are essential for plant defense against pathogens (Biswas et al., 2023; Song et al., 2022). Similarly, MYC transcription factors control jasmonate‐responsive genes, linking them to biotic stress tolerance (Biswas et al., 2023). The TC‐rich repeats and TCA elements activate genes that respond to pathogen attack, further strengthening the plant's defense mechanisms (Diévart & Clark, 2004; Merlot et al., 2001). These elements highlight the crucial role of transcriptional regulation in mediating crown rust resistance genes' responses to biotic and abiotic stresses. The findings provide critical insights into the transcriptional regulatory mechanisms underlying crown rust resistance in oat, identifying potential targets for genetic improvement in breeding programs. The identified CAREs point to a complex network of regulatory pathways that govern plant immunity. Hormonal regulation (via ABA, JA, and GA), light responsiveness, and stress‐responsive elements (such as MYB, MYC, TC‐rich repeats, and TCA elements) collectively activate and fine‐tune defense genes, enabling effective responses to crown rust infection. Understanding these regulatory mechanisms offers valuable insights into the genetic basis of disease resistance, creating opportunities for the development of resistant oat cultivars through molecular breeding and genetic engineering. Integrating these CAREs into functional analyses and breeding strategies could significantly enhance the development of resistant oat cultivars.

### 4.3 MQTL‑assisted breeding for crown rust resistance

MQTL‐assisted breeding primarily focuses on developing improved cultivars with increased disease resistance, with a focus on long‐term durability. MQTL with reduced CIs and those incorporating multiple QTLs have been widely recommended in previous studies for breeding disease‐resistant crops such as wheat (S. Kumar et al., 2023; N. Pal et al., 2022; Saini et al., 2022; Vasistha et al., 2024), rice (Devanna et al., 2024; Goyal et al., 2024; I. S. Kumar & Nadarajah, 2020), maize (M. Gupta et al., 2023; Sunitha et al., 2024), and cotton (Huo et al., 2023). This study selected specific MQTL for potential utilization in breeding programs based on stringent criteria: (i) a CI of less than 1 cM and (ii) the inclusion of at least five initial QTLs. These MQTLs include MQTL_(_ *_Pc_*_)_1D.3, MQTL_(_ *_Pc_*_)_2C.1, MQTL_(_ *_Pc_*_)_2D.1, MQTL_(_ *_Pc_*_)_4A.4, MQTL_(_ *_Pc_*_)_4D.1, MQTL_(_ *_Pc_*_)_5C.3, MQTL_(_ *_Pc_*_)_5D.2, MQTL_(_ *_Pc_*_)_7A.1, and MQTL_(_ *_Pc_*_)_7A.2. The number of initial QTL contributing to these MQTLs ranges from 5 to 16, reflecting their robustness and integration of valuable resistance‐associated loci. Strategic selection of MQTL regions is highly valuable for breeding programs, particularly when they coincide with genes already present in mapped populations. In cases where these genes are absent, MQTL can guide pre‐breeding efforts by identifying resistance‐associated loci for targeted introgression. By consolidating information from multiple QTLs, MQTLs highlight stable genomic regions linked to resistance traits. Molecular markers, such as Kompetitive allele specific PCR markers, developed from these regions, serve as efficient tools for MAS, facilitating the detection and incorporation of favorable alleles while simplifying breeding workflows and reducing costs.

Incorporating well‐characterized MQTL into breeding pipelines enhances genetic gains and promotes stable resistance traits across diverse environments. This strategy underscores the value of MQTL‐assisted breeding in accelerating the development of oat cultivars with durable crown rust resistance. To maximize practical application, it is essential to link MQTL regions to germplasm sources by identifying specific oat lines or populations carrying favorable alleles. This will enable breeding programs to efficiently introduce these resistance loci into new cultivars. The integration of robust genetic information through breeder‐targeted MQTL lays a strong foundation for effective MAS, contributing to reliable trait improvement and long‐term disease management. The comparative analysis of crown rust resistance across *Avena* species reveals significant genetic diversity, indicating opportunities for targeted breeding strategies. Combining previously identified *R* and APR genes with MQTL and candidate genes identified in this study, we have gained a better understanding of the genomic architecture underlying crown rust resistance. Species such as *A*. *sterilis* and *A*. *sativa* are valuable resources due to their multiple resistance genes and associated MQTL. Refined MQTLs, characterized by narrow CIs and enriched with candidate genes, provide precise genomic targets for resistance breeding. These results emphasize the substantial genetic diversity across *Avena* species, positioning *A*. *sterilis* and *A*. *sativa* as key contributors to breeding programs. While seedling resistance genes dominate, APR genes are essential for durable resistance, as their allowance of low levels of disease exerts weaker selection pressure on pathogens, reducing the likelihood of adaptation to resistance (Rimbaud et al., 2023). Identifying MQTL linked to these resistance genes refines genomic regions for targeted breeding efforts. This study underscores the importance of utilizing diverse *Avena* germplasms to enhance crown rust resistance in oat varieties and advance sustainable breeding strategies.

## 5 CONCLUSION

This study represents the first report of MQTL analysis for crown rust resistance in oat, identifying several major genomic regions associated with this critical trait. By combining data from multiple QTL mapping studies, the study gained a better understanding of the genetic architecture of crown rust resistance in the oat genome. A total of 23 reliable MQTLs were identified across 12 chromosomes, with notable examples like MQTL_(_ *_Pc_*_)_1D.3, MQTL_(_ *_Pc_*_)_1D.1, and MQTL_(_ *_Pc_*_)_5D.2, which consolidate a large number of initial QTL, demonstrating their stability and importance for crown rust resistance. The analysis of candidate genes in these MQTLs revealed their involvement in key metabolic pathways, such as polyamine metabolism and amino acid breakdown, which play crucial roles in enhancing plant defense mechanisms. Crown rust resistance appears to be governed by complex regulatory mechanisms, as demonstrated by identifying CAREs associated with hormone signaling, light response, and stress response. These findings have significant implications for oat breeding programs. MQTLs with narrow CIs and many integrated QTL can greatly improve MAS and genomic prediction models. This targeted approach accelerates the breeding process and ensures the development of oat varieties with durable and stable resistance to crown rust across diverse environmental conditions. MQTL and their associated candidate genes for crown rust resistance provide oat breeders with valuable genetic resources for developing cultivars with enhanced crown rust resistance.

## AUTHOR CONTRIBUTIONS

**Nikwan Shariatipour**: Conceptualization; data curation; formal analysis; investigation; methodology; writing—original draft; writing—review and editing. **Mahboobeh Yazdani**: Data curation; investigation; methodology; writing—original draft; writing—review and editing. **Anders Carlsson**: Writing—review and editing. **Therése Bengtsson**: Funding acquisition; writing—review and editing. **Shahryar F. Kianian**: Investigation; writing—original draft; writing—review and editing. **Marja Jalli**: Funding acquisition; writing—review and editing. **Mahbubjon Rahmatov**: Conceptualization; funding acquisition; investigation; methodology; project administration; writing—original draft; writing—review and editing. The manuscript has been shared with all the PPP RobOat Consortium members, who have reviewed it and endorsed it for submission.

## CONFLICT OF INTEREST STATEMENT

The authors declare no conflicts of interest.

## Supporting information




**Table S1** The 1526 putative candidate genes identified in the MQTLs regions.

## Data Availability

All the data generated or analyzed during the current study are included in this published article and its Supporting Information files.
